# Sentiment analysis in multilingual context: Comparative analysis of machine learning and hybrid deep learning models

**DOI:** 10.1016/j.heliyon.2023.e20281

**Published:** 2023-09-19

**Authors:** Rajesh Kumar Das, Mirajul Islam, Md Mahmudul Hasan, Sultana Razia, Mocksidul Hassan, Sharun Akter Khushbu

**Affiliations:** a Department of Computer Science and Engineering, Daffodil International University, Dhaka 1341, Bangladesh; b Faculty of Graduate Studies, Daffodil International University, Dhaka 1341, Bangladesh

**Keywords:** Sentiment analysis, E-commerce, NLP, SVM, Hybrid deep learning, LSTM, Bi-LSTM, Conv1D

## Abstract

This research paper investigates the efficacy of various machine learning models, including deep learning and hybrid models, for text classification in the English and Bangla languages. The study focuses on sentiment analysis of comments from a popular Bengali e-commerce site, "DARAZ," which comprises both Bangla and translated English reviews. The primary objective of this study is to conduct a comparative analysis of various models, evaluating their efficacy in the domain of sentiment analysis. The research methodology includes implementing seven machine learning models and deep learning models, such as Long Short-Term Memory (LSTM), Bidirectional LSTM (Bi-LSTM), Convolutional 1D (Conv1D), and a combined Conv1D-LSTM. Preprocessing techniques are applied to a modified text set to enhance model accuracy. The major conclusion of the study is that Support Vector Machine (SVM) models exhibit superior performance compared to other models, achieving an accuracy of 82.56% for English text sentiment analysis and 86.43% for Bangla text sentiment analysis using the porter stemming algorithm. Additionally, the Bi-LSTM Based Model demonstrates the best performance among the deep learning models, achieving an accuracy of 78.10% for English text and 83.72% for Bangla text using porter stemming. This study signifies significant progress in natural language processing research, particularly for Bangla, by enhancing improved text classification models and methodologies. The results of this research make a significant contribution to the field of sentiment analysis and offer valuable insights for future research and practical applications.

## 1 Introduction

E-commerce platforms have emerged as a widely adopted option for companies, providing them with a diverse array of online purchasing and sales possibilities. These platforms enable consumers to make purchases without having to visit a physical store, unlike regular websites which are often used for information gathering [1]. One such e-commerce website, Daraz, is a popular online shopping marketplace in South Asia, including Bangladesh, Sri Lanka, Pakistan, Myanmar and Nepal. It provides an extensive array of products, featuring over 2.5 million items across diverse categories, including consumer electronics, household necessities, beauty products, fashionable items, groceries, and sports equipment. The global COVID-19 pandemic prompted a notable rise in online purchasing, as nations enforced stay-at-home directives for their residents. As a result of widespread retail closures and concerns about COVID-19 transmission, online shopping has emerged as the dominant method for consumers to fulfill their consumption needs [2].

Bangla is a widely spoken language in Bangladesh and many other countries around the world. As a result, Bangla natural language processing (BNLP) has garnered considerable attention in the field of NLP [3]. Text classification stands as one of the foundational challenges in the field of NLP [4]. However, despite the existence of a large number of E-commerce sites with comment sections allowing for the expression of opinions in the Bengali language, little research has been conducted on Bangla sentiment analysis, which is concerning. English, on the other hand, is widely used in various industries, including search engines, social media, and customer service, making it a crucial language for NLP support. It is also the primary language of many popular online platforms, such as Google, Facebook, and Wikipedia, generating vast amounts of data in English. Additionally, it is the prevailing language employed in scientific publications, making it a focal point for NLP applications in scientific research and knowledge extraction [5,6].

Sentiment analysis is a critical component in assessing opinions on topics such as politics, sports, finances, and product reviews. As humans are subjective, opinions are valuable. E-commerce sites, for instance, are filled with varying perspectives on products, making it essential to detect which statements are positive or negative [7]. Utilizing machine and deep learning algorithms, along with techniques from NLP, it has become feasible to identify instances of cyberbullying and differentiate between bullying and non-bullying statements [8]. Machine learning has become an effective approach for processing data and computation during the past few years, providing smart capabilities in a variety of applications [9]. Algorithms designed for machine learning use statistical, probabilistic, and optimization techniques to draw conclusions from data and identify patterns in unstructured, massive datasets [10]. The potential uses of these algorithms are numerous, including automatic text categorization [11], breast cancer risk prediction [12], machine learning for enhanced data in dermatological image recognition [13], machine learning for speech processing [14], improving medical diagnosis accuracy with causal machine learning [15], statistical arbitrage in cryptocurrency markets using machine learning [16], and classifying fake news [17], among others. Moreover, deep learning algorithms are becoming increasingly significant in different research areas, including but not limited to binary classification-supported multi-class skin lesion classification [18], and analysis of cellular images [19]. User-generated content (UGC) has emerged as a valuable source for understanding consumers' emotions and experiences regarding online retail services. Text mining methods can be utilized to detect various facets within online content, including product information, retailer promotions, delivery services, payment procedures, communication, return/refund policies, and pricing details [20]. With the ongoing advancements in deep learning technology, Convolutional Neural Networks (CNN) and Long Short-Term Memory (LSTM) networks have risen as two of the most influential neural network architectures [21]. With the use of hyperparameter adjustment and a saliency map, CNN models may be constructed directly from infrared spectra [22]. Text classification can be accomplished with the help of machine learning and deep learning methods like Bi-LSTM and CNN models [23], Coordinated CNN-LSTM-Attention (CCLA) models [24], and models for Regional Tree-Structured CNN-LSTM [25], for text sentiment classification and dimensional sentiment analysis. Therefore, these techniques can help analyze user-generated content for insights into consumers' perceptions and experiences.

Based on our literature review, it is evident that the focus of research on machine learning and deep learning algorithms for text analysis has predominantly been on the English language. However, there is a prominent gap in the research that is associated with the Bengali language. This gap in knowledge significantly hinders advancements in areas such as sentiment analysis, NLP, and text classification in Bengali. Consequently, it is crucial to expand research efforts towards analyzing Bengali text data [[26], [27], [28]] and exploring the potential of employing machine learning and deep learning methodologies in this context.

To address this gap, our paper aims to make the following contributions.

1. Dataset Development: We have compiled a comprehensive dataset for sentiment analysis in Bangla by collecting reviews in Bengali and their corresponding English translations. Additionally, we performed data cleaning and preprocessing to ensure the dataset's suitability for analysis.
2. Comparative Analysis: We conducted a comparative analysis that includes traditional machine learning models such as Logistic Regression (LR), Decision Tree (DT), Random Forest (RF), Naive Bayes (NB), K-Nearest Neighbor (KNN), Support Vector Machine Classifier (SVM), Stochastic Gradient Descent (SGD), as well as deep learning-based models. To evaluate the performance of each model, we analyzed various metrics including accuracy, precision, recall, F1-score, and loss metrics from the confusion matrix. Furthermore, we examined the maximum, minimum, and average length of Bengali and English text comments. This analysis provides insights into the effectiveness of both traditional and deep learning methods for text classification in Bengali and English comments.
3. Neural Network Architectures: We designed four unique neural network architectures: Long Short-Term Memory (LSTM), Bidirectional LSTM (Bi-LSTM), Convolutional Neural Network with one-dimensional convolutional layers (Conv1D), and a hybrid architecture combining Conv1D and LSTM layers (Conv1D-LSTM). These architectures were developed to explore their respective capabilities in modeling sequential data, specifically in the context of Bengali text analysis.

The paper's organization is outlined as follows: Section 2 provides an overview of the Literature Review, while Section 3 elaborates on the methodology and materials utilized in the study. Moving forward, Section 4 presents the experimental investigation, encompassing outcomes and performance. In Section 5, Recommendations and Policy Implications are covered, followed by Section 6, which addresses Study Limitations and Scope for Future Research. Lastly, Section 7 provides a summary of the article's conclusion.

## 2 Literature review

Many academic and commercial researchers are currently studying and exploring sentiment analysis [[29], [30], [31], [32]]. In this literature review, we explore various approaches to text sentiment analysis proposed in recent academic research. Liu et al. [33] performed text sentiment classification by employing a bi-LSTM-based structure that incorporates an attention mechanism and a convolutional layer. Similarly, in paper [34], a CNN-based Model was presented for sentiment Classification. In Paper [35], a two-layer, bidirectional LSTM network combined with complicated sentiment analysis units was proposed. In contrast, Du et al. [36] suggested that CNN is a viable model for extracting attention from text and conducted a series of experiments. Kim et al. [37] completed a significant number of experiments on one-layer convolutional neural networks. Chatterjee et al. [38] applied two components – content polarity and overall sentiment content – to accurately depict sentiment found in textual data. In the financial domain, Nelson et al. [39] recommended using LSTM networks with technical analysis indicators to predict stock price. In paper [40], sentiment polarity was identified as an important indication of client happiness, enabling businesses to better understand their customers. In terms of classification approaches, Zhou et al. [41] proposed a standard CNN and LSTM mixed text categorization approach. Alhawarat et al. [42] developed an Arabic text categorization system using CNNs and TF-IDF feature extraction achieving 98.89% accuracy. In language-specific studies, Chowdhury et al. [43] conducted sentiment analysis on 4000 manually translated Bangla movie reviews achieving 82.42% accuracy with LSTM. Chakraborty et al. [44] used machine learning to predict feature ratings and diagnostics from text, while Zhang et al. [45] presented a CNN for text classification at the character level that significantly improved accuracy. In paper [46], a unique method using differential privacy was proposed to improve the LSTM model's stock prediction capabilities. Paper [47] performed a comparative analysis for classifying sentiment in Bangla news comments using both traditional SVM and deep learning (LSTM and CNN) algorithms. Paper [48] compared the performance of back-propagation-based neural networks for text classification when compared to other supervised machine learning models. Overall, these studies offer valuable insights into the development and application of various neural network (NN)-based models for text sentiment analysis and classification.

## 3 Data and methodology

**Fig. 1:**
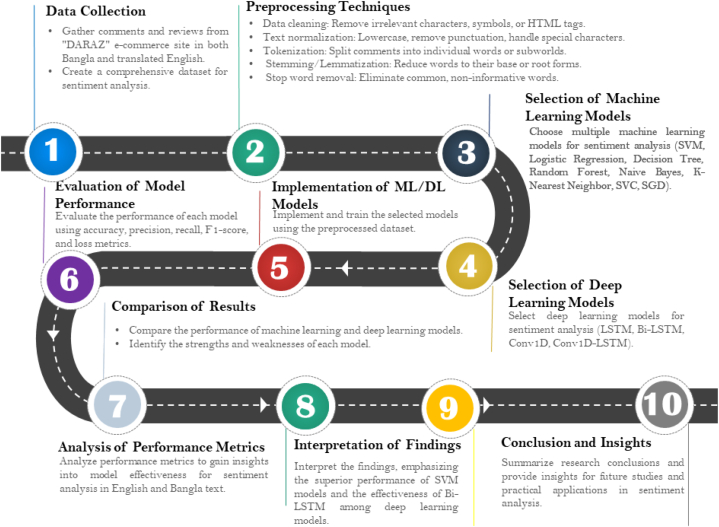
Roadmap of proposed text classification methodology.

The methodology utilized in this research comprises multiple phases, including data collection, data preprocessing, model selection, statistical assessment, and implementation, all of which are depicted in Fig. 1.

### 3.1 Dataset preparation

The preparation of a dataset for Natural Language Processing (NLP) typically involves several stages, including data collection and curation, classification into distinct categories, pre-processing and cleaning, as well as splitting the data into training, annotation, balancing, validation, and test sets. This process entails organizing and cleaning the data to make it suitable for use in NLP tasks. The subsequent step involves selecting an appropriate algorithm for the task at hand. The model's performance is evaluated using statistical analysis, and the final step involves deploying the model and monitoring its performance in real-world settings. Notably, the dataset preparation process involves two key stages which are Data Collection and Preprocessing of the Data.

### 3.2 Data collection

We have assembled a dataset consisting of 2577 reviews in Bengali, along with their corresponding English translations and class labels. The dataset was collected from the Daraz E-commerce site using web scraping techniques. The reviews in the dataset encompass both positive and negative comments and have been carefully curated specifically for the purposes of this study. Table 1 presents an example of collected texts for sentiment analysis in both Bangla and English.

**Table 1:** Sample collected texts for sentiment analysis in Bengali and English.

Bangla Review	English Review (Translated)	Sentiment
আমার জীবনে দেখা সবচাইতে খারাপ পণ্য	The worst product I've ever seen	Negative
ভালো লাগলো আন্তর্জাতিক ব্র্যান্ড গুলো এখন বাংলাদেশেই পাওয়া যাচ্ছে	Good international brands are now available in Bangladesh	Positive
মানুষকে না ঠকিয়ে ভাল প্রোডাক্ট দেওয়ার ব্যবস্থা করেন	Provide good products without deceiving people	Negative
দাম অনুযায়ী এই পণ্য পাওয়া ভাগ্যের ব্যাপার।	It's a matter of luck to get this product by price	Positive
এতো বাজে কমেন্টস দেখে কেনার সাহস হারিয়ে ফেলেছি	I lost the courage to see so many bad comments	Negative

**Fig. 2:**
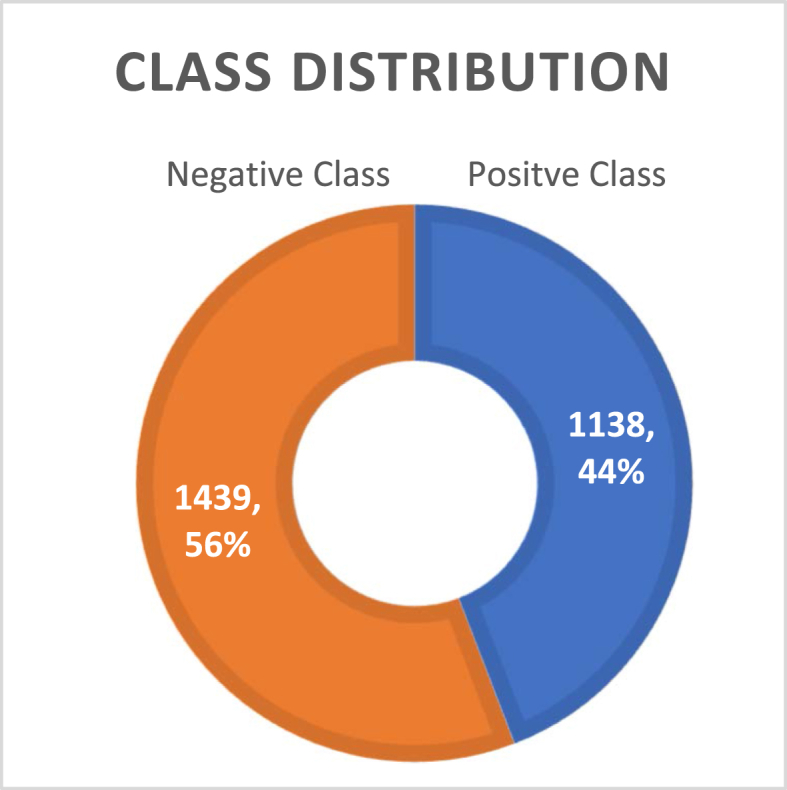
Dataset class distribution overview (English and Bangla both).

Following data collection, the dataset underwent manual annotation to ensure the accuracy and consistency of class labels. Subsequently, the data was preprocessed to remove irrelevant information, correct errors, and ensure compatibility with the Natural Language Processing (NLP) model. This preprocessing step is essential for improving the quality of the dataset and enhancing the performance of the NLP model during training and evaluation. As depicted in Fig. 2, the dataset for English and Bangla text contained a total of 1439 positive comments and 1138 negative comments.

### 3.3 Data preprocessing

**Fig. 3:**
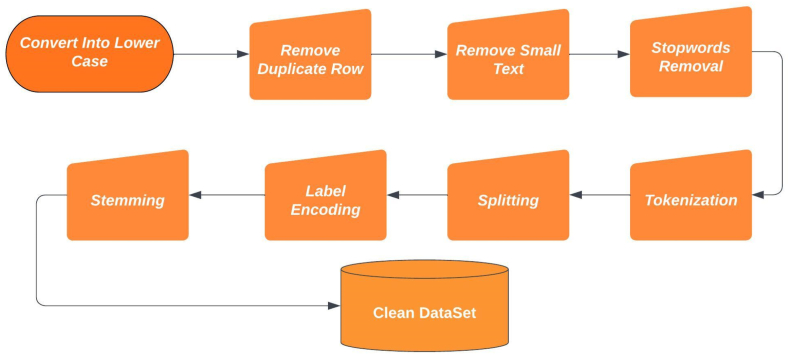
Data preprocessing steps.

Data preprocessing plays a vital role in readying data for Natural Language Processing (NLP) tasks. In NLP, data preprocessing involves the process of cleaning text to remove irrelevant information, correcting errors, tokenizing text into words, phrases, or sentences, stemming and lemmatization are processes that strip words down to their simplest components, identifying and removing any non-representative data points, and normalizing data to ensure consistency. These steps aid in enhancing the accuracy and efficiency of NLP algorithms. In this study, we present our contribution to preprocessing English and Bangla comments data, as illustrated in Fig. 3. The preprocessing steps involved in our study include cleaning the text to remove irrelevant information and errors, tokenizing the text into words and sentences, stemming the words to their base form, identifying and removing any non-representative data points, and normalizing the data to ensure consistency.

#### 3.3.1 Convert into lower case

Text normalization is a common preprocessing step in sentiment analysis for English text, whereby all characters are converted to lowercase. However, for Bangla text, this process is unnecessary. In our study, we converted all words to lowercase for the English language to simplify the data and facilitate processing and sentiment analysis by machine learning models. Capitalization variations in sentiment analysis datasets can hinder accurate sentiment identification by algorithms. Lowercasing eliminates such variations and generates a more consistent dataset. Furthermore, reducing the number of unique tokens through this process leads to increased computational efficiency in analysis and model training, resulting in more effective sentiment analysis. By reducing the variations in text and improving the consistency of the data, preprocessing steps such as lowercasing enable machine learning models to more accurately identify and classify sentiment.

#### 3.3.2 Removing duplicate rows

The removal of duplicate rows is a crucial step in data preprocessing for NLP tasks since they can lead to inaccuracies in models and increased processing time. In this study, we removed duplicate rows from the English and Bangla datasets. This process involves identifying the duplicate rows, selecting a strategy for removing them, such as keeping the first instance or the one with the highest confidence score and implementing the strategy using a programming language or tool. By eliminating duplicate rows, we aimed to enhance the accuracy of NLP models and reduce processing time. This step contributes to the quality of the dataset and the overall effectiveness of sentiment analysis. Therefore, we removed duplicate rows for both English and Bangla datasets to ensure the reliability of the results obtained from our analysis.

#### 3.3.3 Removing small texts

Filtering small texts, or texts that are below a certain length is an important step in preprocessing data for NLP tasks. Such texts, including individual words or brief sentences, may not carry enough information and thus can be removed to improve the quality of the dataset. The process involves determining the minimum length of texts to be kept in the dataset, identifying texts below the threshold, and utilizing a programming language or software tool to remove them. This approach has the potential to enhance the precision of NLP models, and the quality of the dataset can be enhanced. In our study, we determined the length of the sentence for both English and Bangla. We then provided the minimum length of texts to be kept in the dataset. After cleaning small texts, we removed one small conversation for English sentences and one small conversation for Bangla sentences.

#### 3.3.4 Stopwords removal

Stopwords removal is a frequently used preprocessing technique in NLP tasks. Stopwords are words such as "the," "a," and "and" that do not carry much meaning and can be removed to simplify the dataset and speed up processing. The process involves identifying stop words, determining a strategy for removal such as removing all or only the most frequently occurring, and using a programming language or software tool to execute the strategy. This helps to make the dataset smaller and processing faster, potentially enhancing the accuracy of NLP models. For the English dataset, we cleaned words like - {“about”, “don't”, “across”, “after”, “again”, “your”, “me”, “not”} etc. For Bangla dataset, we cleaned word like - {“এই”, “কোন”, “আমি”, “আপনার”, “যা”, “যে”, “মনে”, “করি”} etc.

#### 3.3.5 Tokenization

NLP tokenization is a key process that involves dividing text into individual words or phrases known as tokens. The tokenization process is a critical step in preprocessing data for NLP tasks such as analysis of sentiment, classification text, and machine translation. Tokenization helps to standardize the text data by breaking it down into smaller units that are easier to process and analyze. In our code, we used the Tokenizer class from Keras to tokenize the sentences. The core steps of tokenization were: Define the maximum number of features (words) to consider, create an instance of the Tokenizer class, set the maximum number of words to be considered, specify the split character as a space, fit the tokenizer on the text data, convert the text sentences to sequences of integers, pad the sequences to ensure uniform length. These steps allowed us to transform the sentences into numerical representations that could be processed by our deep learning model.

#### 3.3.6 Punctuation, special character and number removal

The dataset has been preprocessed to remove punctuation marks such as ।, ., ? !, and special characters like @, #, $, %, ^, &, *, etc., as well as numbers that are not important for sentiment analysis.

#### 3.3.7 Splitting

Splitting is a fundamental technique in NLP that involves dividing text into smaller, meaningful units for analysis. It is a critical preprocessing step in NLP tasks as it enables the processing of text data by machine learning models. Text splitting can be performed at various levels, such as the word, sentence, or paragraph level. At the word level, the text is divided into individual words, also known as tokens, by a process called tokenization.

#### 3.3.8 Categorical encoding

Categorical encoding is a process in NLP where categorical variables are converted into numerical values that can be used as inputs for machine learning models. Categorical variables are those that have a limited number of possible values, such as words in a vocabulary or items on a menu. Categorical encoding comes in two forms: label encoding and embedding. Label encoding is a way of converting categorical data into numerical format by assigning integer values to each unique category. In our study, label encoding provides a means of transforming categorical data, which cannot be processed directly, into a numerical form. Label encoding assigns each category a unique integer value. The integer values are assigned without implying any ranking or order between the categories. On the other hand, Embedding is a method applied in NLP and machine learning to convert data into dense, low-dimensional vectors. For NLP, words or phrases are transformed into word embeddings, which are high-dimensional vectors that depict their semantic significance and the relationships between words. Word embedding methods rely on the principle that words with similar meanings tend to be used in similar contexts. For example, in our English comment dataset, “good” and “best” would likely be found in similar contexts and thus have similar meanings. For example, in our Bangla comment dataset, "ইয়ারফোন", "হেডসেট" and "হেডফোন" would likely be found in relevant contexts and thus have relevant meanings also. The word embeddings of these words would be alike, reflecting the relationships between them. Embedding enhances NLP model performance by encapsulating word meaning and word relationships in a dense, low-dimensional format.

#### 3.3.9 Stemming

Stemming is a technique widely used in NLP to simplify words to their base form. Its primary objective is to simplify of the text dataset, making it easier to analyze. In our study, we have applied both the Lancaster and Porter Stemming Algorithms to our models. The purpose of stemming is to transform words into their common base form. For instance, in English texts, words such as "recommended," "recommends," and "recommendation" would be transformed to "recommend" through stemming. Similarly, in Bangla texts, words like “করেছিলাম” and “করছে” would be transformed to “করেছি”. It involves extracting words, applying the chosen algorithm, and storing the stemmed words for analysis. Stemming improves NLP model accuracy but can sacrifice information and interpretability. We considered this trade-off while applying stemming algorithms to our models.

### 3.4 Dataset summary

**Fig. 4:**
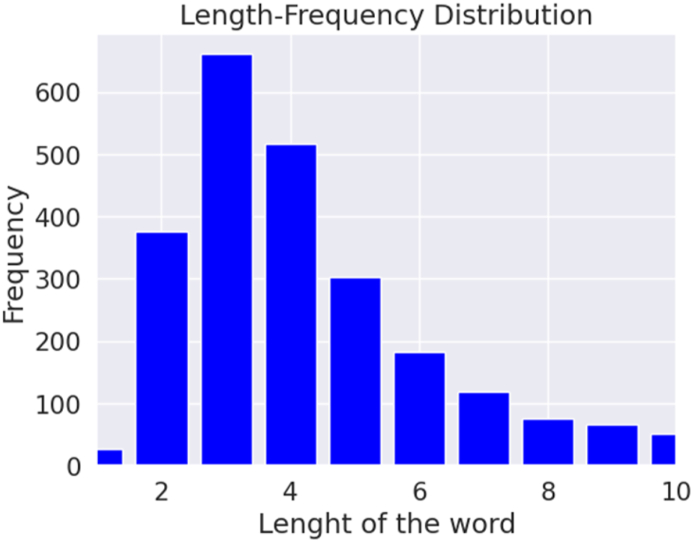
Length of the word for English.

**Fig. 5:**
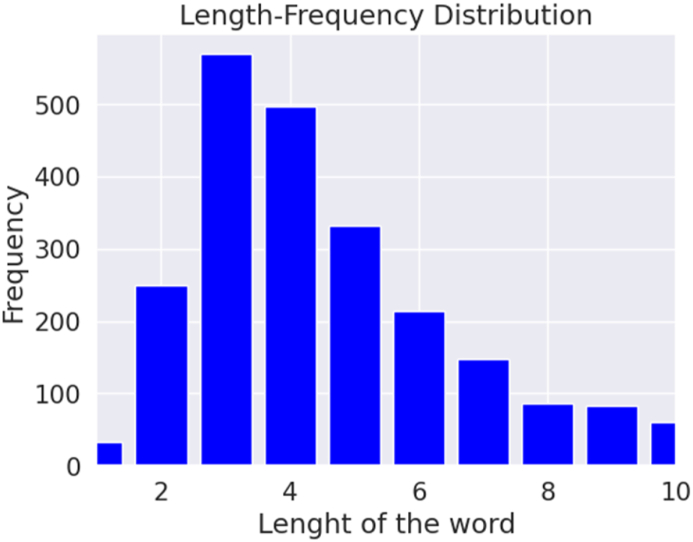
Length of the word for Bangla.

**Fig. 6:**
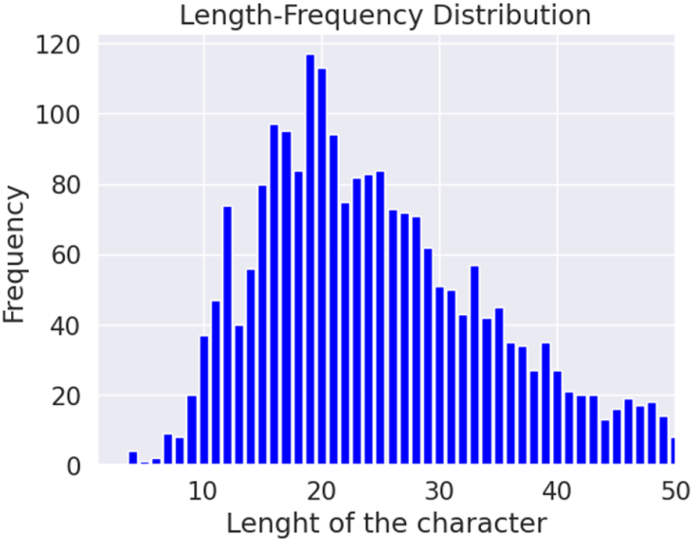
Length of character for English texts.

**Fig. 7:**
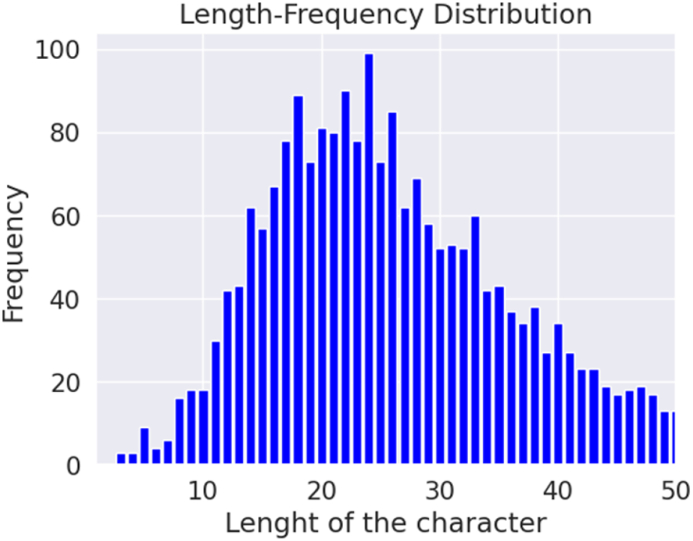
Length of character for Bangla texts.

Our summary entails various details such as the total comments, the distribution of data across different classes, the number of words, the number of unique words, most frequent words, average length, maximum length, and a minimum length of comments. Understanding the characteristics of the dataset is crucial for selecting appropriate models and techniques for an NLP task and comprehending and evaluating the results of model evaluations. Fig. 4, Fig. 5 depict the length-frequency distribution for English and Bangla texts for the length of the word, providing a clear visual representation. Furthermore, Fig. 6, Fig. 7 provide a clear visual representation of the length-frequency distribution for English and Bangla texts for the length of the character.

### 3.5 Machine learning algorithms and statistical analysis

The dataset underwent division utilizing the train-test split technique, whereby the larger portion of the data (80%) was designated for training the model, with the remaining 20% utilized for testing. This procedure was applied to both English and Bangla texts. This approach is commonly employed in machine learning to evaluate the performance of models on unseen data. The model is trained with the help of the training data, while the performance of the model in predicting the outcomes of new data is evaluated with the help of the test data. Such an approach aids in detecting overfitting, This is a standard issue in machine learning. Our study employed several supervised machine learning algorithms, including Support Vector Machine, Multinomial Naive Bayes, K-Nearest Neighbors, Logistic Regression, Decision Tree, Random Forest, and Stochastic Gradient, to assess their performance in sentiment analysis.

#### 3.5.1 Feature extraction

In the field of natural language processing, machine learning techniques are used for accomplishing diverse goals. One such technique is tokenization, which involves breaking down phrases into individual word components. These components, whether common or unique, are then analyzed for specific characteristics. Another crucial technique is TF-IDF, a numerical metric that evaluates the importance of specific terms within a text. This approach has been widely utilized by reputable publications in multiple languages and has been proven effective. Our study was inspired by these successful methods, and we have discovered that our learning algorithms attain high accuracy when employing them.

#### 3.5.2 Data vectorization or distribution

The Count Vectorizer, part of the helpful Python Scikit-learn toolkit, takes into consideration the frequency of each word in the text to convert a phrase into a vector. The size of the n-grams used can be specified using the ngram_range parameter. As an illustration, setting the value to 1, 1 would produce unigrams (n-grams consisting of a single word), whereas a value of 1–3 would yield n-grams ranging from one to three words. Table 2 and Table 3 display examples of n-gram distribution for both the English and Bangla languages.

• Unigram: Setting n = 1 in the n-grams function generates unigrams or 1-g, allowing for word frequency calculations.
• Bigram: Configuring n = 2 in the n-grams function generates bigrams or 2-g, facilitating word frequency calculations.
• Trigram: Specifying n = 3 in the n-grams function generates trigrams or 3-g, enabling word frequency calculations.

**Table 2:** Example of n-gram distribution for English.

Sentence	Uni-gram	Bi-gram	Tri-grams
This is the best phone in the budget.	('the', 2), ('this', 1), ('is', 1)	('this is', 1), ('is the', 1), ('the best', 1)	('this is the', 1), ('is the best’, 1), ('the best phone', 1)

**Table 3:** Example of n-gram distribution for Bangla.

Sentence	Uni-gram	Bi-gram	Tri-grams
**আমি মনে করি আমি আমার টাকা অপচয়(I think I wasted my money)**	**('আম', 3),** **('মন', 1),** **('কর', 1)**	**('আম মন', 1),** **('মন কর', 1),** **('কর আম', 1)**	**('আম মন কর', 1),** **('মন কর আম', 1),** **('কর আম আম', 1)**

### 3.6 DNN-based models

DNN, which stands for Deep Neural Network, represents a category of artificial neural networks characterized by multiple layers, including hidden layers, additionally to the layers for input and output. Networks with these concealed layers enable the learning and representation of intricate data patterns and relationships, rendering DNNs valuable for tasks like high-dimensional data involving image and speech recognition, natural language processing, and more. In the current study, we designed and implemented four unique NN models, namely LSTM, Bi-LSTM, Convolutional Neural Network with one-dimensional convolutional layers (Conv1D), and a hybrid architecture comprising Conv1D and LSTM layers (Conv1D-LSTM). Table 4 illustrates the experimental setup of four Deep Neural Network (DNN) models.

**Table 4:** Experimental setup of four Deep Neural Network (DNN) models.

Model Name	Embedding Layer	Conv1D Layer	LSTM Layer	Bi-LSTM Layer	Fully Connected Layer	Dropout Layer	Classification Layer	Batch Size	Epoch
LSTM Based Model	64	N/A	Layer: 1 Unit: 64	N/A	Layer: 1 Unit: 256	Layer: 1 (40%)	Softmax	32	50
Bi-LSTM Based Model	64	N/A	N/A	Layer: 1 Unit: 64	Layer: 1 Unit: 256	Layer: 1 (40%)	Softmax	32	50
Conv1D Based Model (CNN)	64	Layer: 128 Unit: 5	N/A	N/A	Layer: 1 Unit: 256	Layer: 1 (40%)	Softmax	32	50
Combine Conv1D & LSTM Based Model	64	Layer: 128 Unit: 5	Layer: 1 Unit: 64	N/A	Layer: 1 Unit: 256	Layer: 1 (40%)	Softmax	32	50

#### 3.6.1 LSTM (Long Short-Term Memory)

**Fig. 8:**
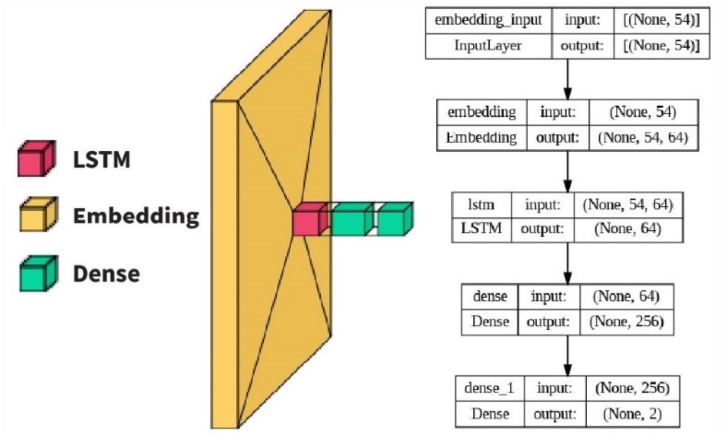
LSTM-based model.

The proposed LSTM model shown in Fig. 8 is a sequential model, commonly used for sequential data processing such as time series, speech, and text. It consists of the following layers: Embedding Layer: This layer converts the input features into dense vectors of fixed size (embed dim) and allows the model to learn representations specific to the input data. The input length parameter is set to the number of features in the input. LSTM Layer: This layer utilizes LSTM cells to process the sequential data. The LSTM layer has 64 units/neurons and is configured with a dropout rate of 0.2 to regularize the network and prevent overfitting. The recurrent dropout parameter is set to 0.4, indicating that dropout is applied to the recurrent connections within the LSTM cells. Dense Layer: A fully connected layer with 256 units and a SoftMax activation function. This layer helps to transform the LSTM layer's output into a format suitable for the final classification task. Output Layer: Another dense layer with 2 units and a sigmoid activation function. This layer produces the final output probabilities for the binary classification task. The model is compiled with the binary cross-entropy loss function, the Adam optimizer, and the accuracy metric. The model summary shows that there are a total of 82,178 parameters in the model, all of which are trainable.

#### 3.6.2 Bi-LSTM (bidirectional Long Short-Term Memory)

**Fig. 9:**
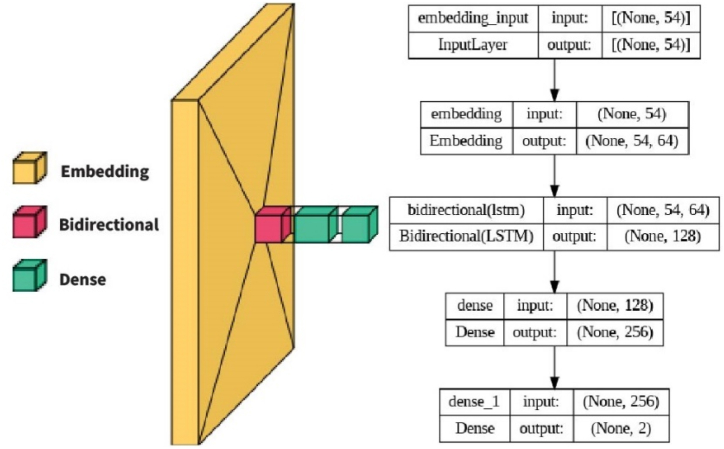
Bi-LSTM based model.

The Bi-LSTM model is a recurrent neural network that utilizes two LSTM models, one processing the input sequence forward and the other backward, to capture both past and future context shown in Fig. 9. The proposed Bi-LSTM model is a sequential model that includes the following layers: Embedding Layer: Converts input features into dense vectors of size embed dim for learning representations specific to the input data. Bidirectional LSTM Layer: Utilizes bidirectional LSTM cells to process the sequential data in both forward and backward directions. It has 64 units/neurons, a dropout rate of 0.2 for regularization, and a recurrent dropout rate of 0.4 for dropout on the recurrent connections. Dense Layer: A fully connected layer with 256 units and SoftMax activation, transforming the output of the Bidirectional LSTM layer for the classification task. Output Layer: Another dense layer with 2 units and sigmoid activation, producing the final output probabilities for binary classification. The model is compiled with binary cross-entropy loss, Adam optimizer, and accuracy metric. It has a total of 131,586 trainable parameters.

#### 3.6.3 CNN (convolutional neural network)

**Fig. 10:**
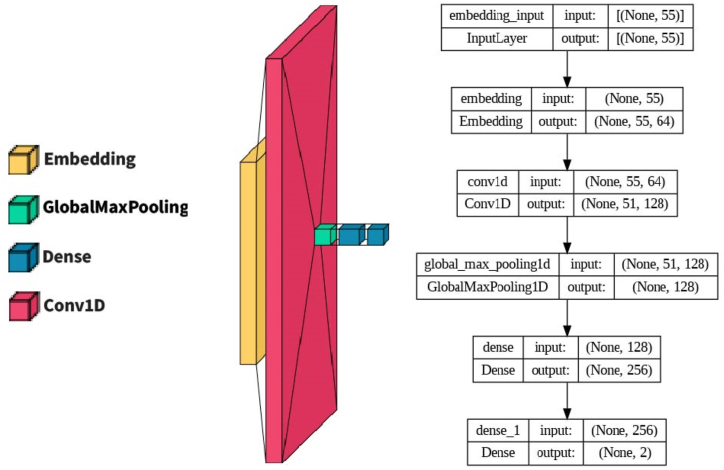
Conv1D-based model (CNN).

Convolutional Neural Networks (CNNs) are a type of neural network commonly used in image recognition and computer vision tasks. They consist of multiple layers of interconnected nodes, where each node performs a convolution operation on a subset of the input data. The proposed CNN model illustrated in Fig. 10 consists of the following layers: Embedding Layer: This layer converts input features into dense vectors of size embed dim to capture meaningful representations specific to the input data. Conv1D Layer: This layer performs a convolution operation on the input data using 128 filters, each with a size of 5. The activation function used is ReLU, which introduces non-linearity into the network. GlobalMaxPooling1D Layer: This layer takes the maximum value across the temporal dimension of the convolved features, reducing the dimensionality of the data. Dense Layer: A fully connected layer with 256 units and SoftMax activation. It transforms the pooled features into a format suitable for the final classification task. Output Layer: Another dense layer with 2 units and sigmoid activation, producing the final output probabilities for binary classification. The model is compiled with binary cross-entropy loss, Adam optimizer, and accuracy metric. It has a total of 106,626 trainable parameters.

#### 3.6.4 Hybrid Conv1D-LSTM

**Fig. 11:**
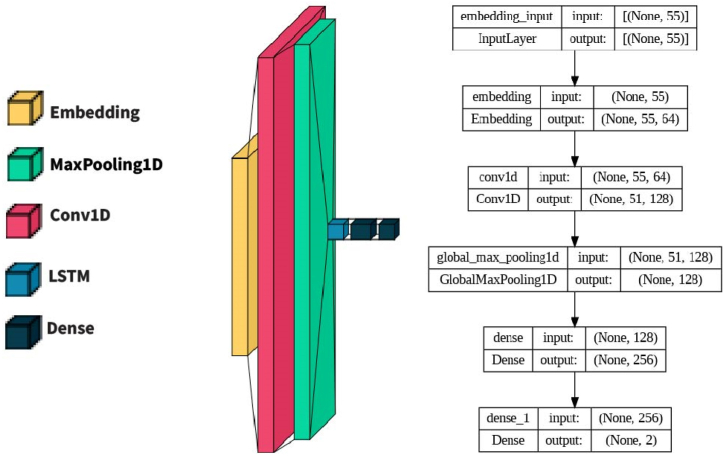
Combined Conv1D and LSTM-based model.

Both CNNs and LSTMs are often used in combination with other types of DNNs to improve performance on various tasks. The architecture mentioned in Fig. 11 is commonly employed in tasks that involve analyzing sequential data, such as natural language processing and speech recognition. The proposed Hybrid Conv1D-LSTM model combines Conv1D and LSTM layers to process sequential data. The architecture is as follows: Embedding Layer: Converts input features into dense vectors of size embed dim to capture meaningful representations specific to the input data. Conv1D Layer: Performs a 1D convolution operation on the input sequence using filters and a kernel size. The activation function used is ReLU. MaxPooling1D Layer: Reduces the dimensionality of the convolved features by taking the maximum value across the temporal dimension. LSTM Layer: Processes the features from the Conv1D layer using LSTM cells with 64 units. It includes dropout regularization with a dropout rate of 0.2 and recurrent dropout rate of 0.4. Dense Layer: A fully connected layer with 256 units and SoftMax activation, transforming the LSTM layer's output into a format suitable for the classification task. Output Layer: Another dense layer with 2 units and sigmoid activation, producing the final output probabilities for binary classification. The model is compiled with binary cross-entropy loss, Adam optimizer, and accuracy metric. It has a total of 139,650 trainable parameters.

### 3.7 Experimental setup

**Fig. 12:**
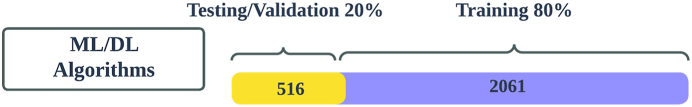
Data distribution.

The present experiment was conducted utilizing the Google Colab platform for the purpose of training learning models. This platform offers unrestricted access to high-performance Graphics Processing Units (GPUs) with minimal setup requirements. To assess the performance of the models, a train-test split was employed. Specifically, an 80-20 split was utilized, allocating 80% of the data samples for model training and the remaining 20% for model testing shown in Fig. 12. This division ensures an unbiased evaluation of the models' capabilities. During the training process, key hyperparameters were set as follows: the number of epochs was set to 50, with a batch size of 32 for efficient optimization. To provide insights into the training progress, the verbose level was set to 1, enabling the display of training updates and performance metrics.

## 4 Result and discussion

The Results and Discussion section offers a comprehensive overview of the empirical findings and their corresponding interpretations. Within this section, a detailed analysis of the acquired data is provided, encompassing pertinent performance evaluation metrics such as accuracy, precision, recall, and F1-score. Furthermore, an exploration of the influence exerted by various parameters and hyperparameters on the model's performance is undertaken. Notably, a comparative assessment is conducted, contrasting the outcomes derived from conventional machine learning models against those emanating from deep learning models.

Table 5, Table 6 provides a comparison of different models used for stemming in natural language processing tasks. It includes the model names, stemming algorithm used (Lancaster or Porter), and the performance metrics: accuracy, precision, recall, and F1 score for English and Bangla text respectively.

**Table 5:** Performance score of ML models for English text.

Model Name	Stemming Algorithm	Accuracy (%)	Precision (%)	Recall (%)	F1 Score (%)
Logistic Regression	Lancaster	80.04	79.32	88.01	83.44
	Porter	81.20	77.64	91.79	84.12
Decision Tree	Lancaster	76.71	79.93	79.11	79.52
	Porter	78.88	80.00	81.43	80.71
Random Forest	Lancaster	78.08	82.61	78.08	80.28
	Porter	82.36	83.16	84.64	83.89
Multi Naïve Bayes	Lancaster	80.82	82.77	83.90	83.33
	Porter	81.59	81.79	85.00	83.36
KNN	Lancaster	73.97	77.32	77.05	77.19
	Porter	75.00	74.92	81.07	77.87
SVM	Lancaster	79.26	81.00	83.22	82.09
	Porter	82.56	82.09	86.79	84.37
SGD	Lancaster	80.43	81.37	85.27	83.28
	Porter	81.40	79.87	87.86	83.67

**Table 6:** Performance score of ML models for Bangla text.

Model Name	Stemming Algorithm	Accuracy (%)	Precision (%)	Recall (%)	F1 Score (%)
Logistic Regression	Lancaster	82.95	77.75	96.07	85.94
	Porter	82.95	77.75	96.07	85.94
Decision Tree	Lancaster	82.56	85.19	82.14	83.64
	Porter	82.36	85.13	81.79	83.42
Random Forest	Lancaster	82.36	79.08	91.79	84.96
	Porter	81.01	76.61	93.57	84.24
Multi Naïve Bayes	Lancaster	84.30	83.96	87.86	85.86
	Porter	84.30	83.96	87.86	85.86
KNN	Lancaster	77.91	78.82	81.07	79.93
	Porter	77.91	78.82	81.07	79.93
SVM	Lancaster	86.43	85.71	90.00	87.80
	Porter	86.43	85.71	90.00	87.80
SGD	Lancaster	84.30	83.28	88.93	86.01
	Porter	84.50	85.21	86.43	85.82

In the field of English text analysis, a comparison was made between different models using a dataset, as shown in Table 5. The model that demonstrated the highest accuracy was the SVM (Support Vector Machine) algorithm combined with the Porter stemming algorithm, achieving an impressive accuracy of 82.56%. SVM works by identifying the optimal hyperplane that separates the data into distinct classes, while maximizing the margin between them. On the other hand, the KNN (K-Nearest Neighbors) model with the Lancaster stemming algorithm exhibited the lowest performance, with an accuracy of 73.97%. KNN assigns a class label to a given instance based on the majority vote of its k nearest neighbors. In the case of Bangla text analysis, a similar evaluation was conducted and documented in Table 6. Among the models tested, SVM stood out as the top performer, utilizing both the Porter and Lancaster stemming algorithms. It achieved an impressive accuracy rate of 86.43%. In contrast, regardless of the stemming algorithm used (Porter or Lancaster), the KNN model fell behind in terms of performance, achieving an accuracy of 77.91%.

Table 7 presents the performance scores of various deep learning models for English text analysis. Among the models evaluated, there was a standout performer as well as a model with the poorest performance. The best-performing model in terms of overall performance was the Bi-LSTM based model with the Porter stemming algorithm. This model achieved exceptional results, showcasing an accuracy of 78.10%. The poorest performance was the Conv1D based model with the Lancaster stemming algorithm. This model demonstrated lower scores compared to the other models, with an accuracy of 72.86%.

**Table 7:** Performance score of DL models for English text.

Model Name	Stemming Algorithm	Accuracy (%)	Precision (%)	Recall (%)	F1 Score (%)
LSTM Based Model	Lancaster	77.51	74.45	74.45	74.45
	Porter	77.13	73.39	75.33	74.35
Bi-LSTM Based Model	Lancaster	77.71	74.35	75.33	74.84
	Porter	78.10	75.00	75.33	75.16
Conv1D Based Model	Lancaster	72.86	68.35	71.37	69.83
	Porter	74.22	70.61	70.93	70.77
Conv1D–LSTM Based Model	Lancaster	73.64	70.59	68.72	69.64
	Porter	77.32	73.50	75.77	74.62

Table 8 presents a comprehensive overview of the performance scores achieved by various Deep Learning models when applied to Bangla text analysis, using different stemming algorithms. Notably, the Bi-LSTM based model combined with the Porter stemming algorithm emerged as the top performer among the DL models. This model exhibited an impressive accuracy of 83.72%, indicating its strong proficiency in accurately classifying and predicting Bangla text data. In contrast, the Conv1D based model utilizing the Lancaster stemming algorithm demonstrated the poorest performance in the evaluation. With an accuracy of 71.70%, precision of 71.66%, recall of 59.03%, and an F1 score of 64.73%, this model faced challenges in effectively categorizing Bangla text data. Therefore, further refinement and alternative techniques may be necessary to improve its performance.

**Table 8:** Performance score of DL models for Bangla text.

Model Name	Stemming Algorithm	Accuracy (%)	Precision (%)	Recall (%)	F1 Score (%)
LSTM Based Model	Lancaster	83.52	79.83	83.70	81.72
	Porter	82.94	79.08	83.26	81.12
Bi-LSTM Based Model	Lancaster	82.94	79.32	82.82	81.03
	Porter	83.72	84.21	77.53	80.73
Conv1D Based Model	Lancaster	71.70	71.66	59.03	64.73
	Porter	81.78	78.54	80.62	79.57
Conv1D–LSTM Based Model	Lancaster	73.06	67.74	74.01	70.74
	Porter	72.48	66.28	76.21	70.90

**Fig. 13:**
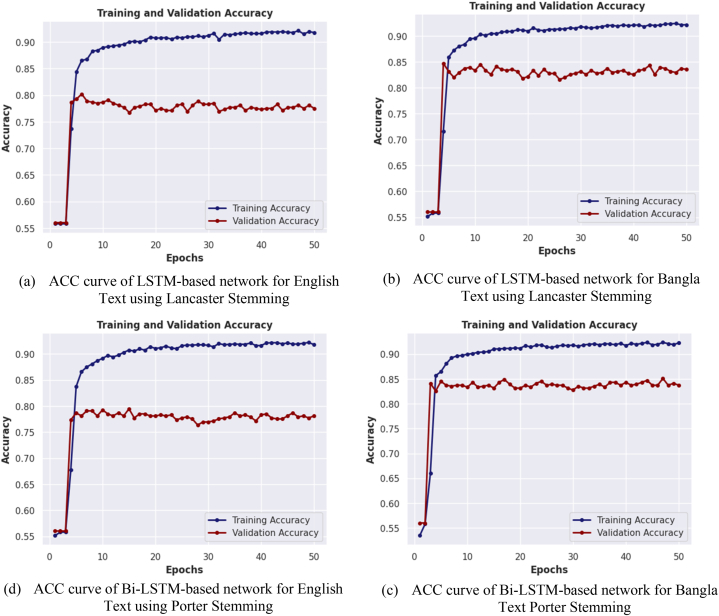

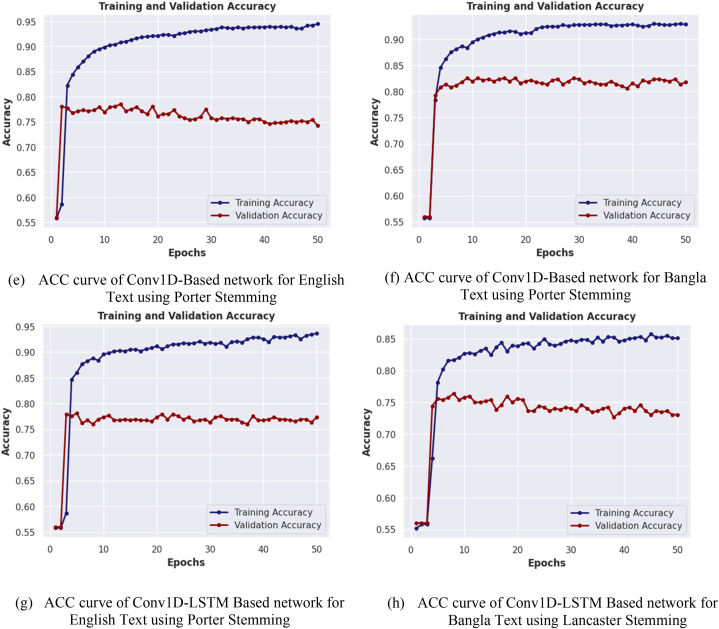
(a–h) Illustrating the accuracy of each network for training and validation datasets over epochs.

In Fig. 13(a–h), graphical comparisons of four distinct models, namely the LSTM-Based Model, Bi-LSTM Based Model, Conv1D Based Model, and combined Conv1D-LSTM Based Model, are depicted for English and Bangla text classification using both the Porter and Lancaster stemming algorithms. These comparisons focus on the accuracy curve of the best performing model for each stemming algorithm. The relationship between the accuracy of each model on both training and validation datasets and the number of training epochs is illustrated in the figure. The results indicate that the accuracy of the models generally increases with the number of training epochs until it reaches a plateau, indicating that additional epochs do not significantly improve the accuracy of the models. Additionally, the figure shows that the rate of accuracy improvement varies across the different models and languages, the figure also highlights the general trend of lower accuracy on the validation dataset compared to the training dataset, indicating a possible overfitting of the models to the training data. It provides valuable insights into the performance of different neural network models for Bangla and English text classification. The results underscore the importance of carefully selecting appropriate models and balancing the number of training epochs to achieve optimal accuracy on both training and validation datasets.

**Fig. 14:**
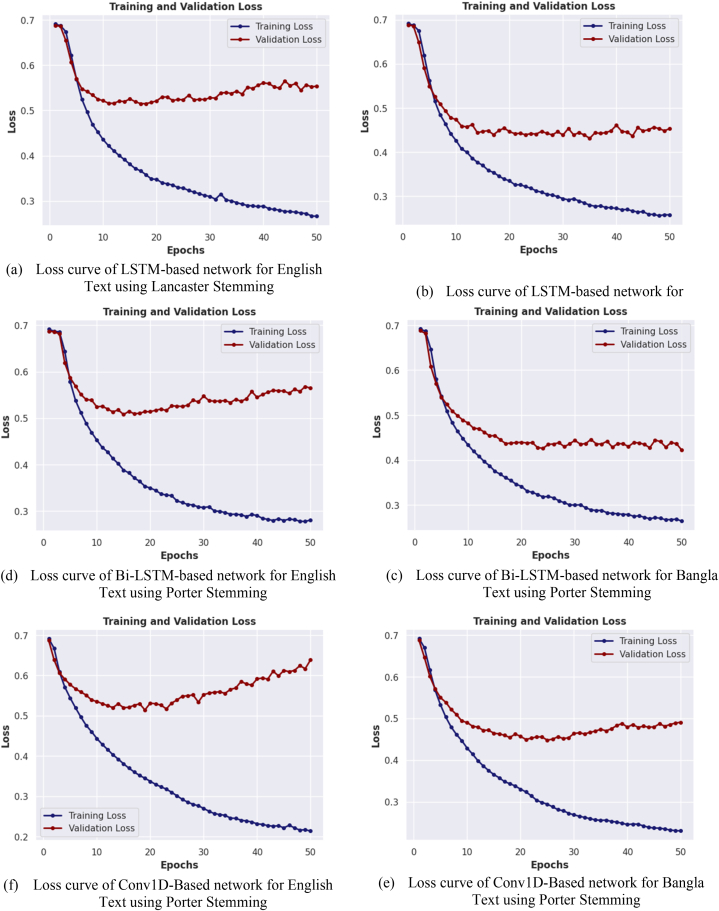

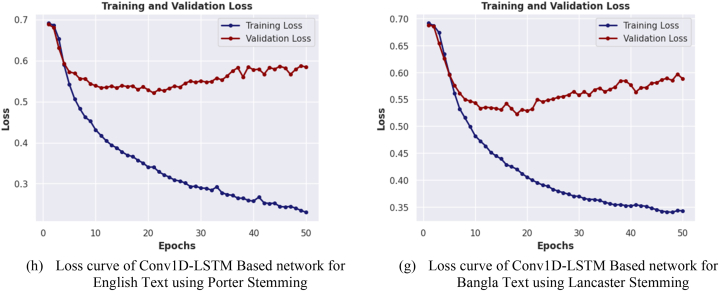
(a–h)) Illustrating the loss of each network for training and validation datasets over epochs.

In Fig. 14(a–h), a graphical illustration is presented, showcasing the relationship between the number of training epochs and the loss of four distinct neural network models for both English and Bangla text classification. The models considered in this comparison utilize both the Porter and Lancaster stemming algorithms. The focus of this analysis is on the loss curve of the best performing model for each stemming algorithm. The figure shows how the loss of the models changes with the number of epochs during the training process. Specifically, the figure indicates that the loss of the models generally decreases with the number of training epochs until it reaches a plateau, indicating that additional epochs do not significantly improve the loss of the models. Additionally, the figure reveals that the loss of the models on the validation dataset is generally higher than that on the training dataset, which suggests the presence of overfitting. This observation highlights the importance of using appropriate validation strategies to evaluate the generalization ability of the models.

The pseudo code provided in Table 9 outlines the step-by-step procedure for implementing SVM in sentiment prediction tasks on external Bangla and English text. The inclusion of SVM in the table is based on its remarkable accuracy, which outperformed all other models evaluated in the study. It serves as a guide for developers and researchers interested in utilizing SVM for sentiment analysis, leveraging its superior accuracy compared to other models examined in the study.

**Table 9:** Pseudo code of Support Vector Machine for sentiment prediction on external Bangla and English Text.

StepDescription	
1	Start.
2	Import the necessary libraries
3	Read the dataset from the input source
4	Preprocess the dataset:
4.1	Convert the sentiment labels to numerical values.
4.2	Apply stemming to the comments.
5	Split the dataset into training and testing sets using train_test_split function.
6	Vectorize the text data using TF-IDF:
6.1	Initialize a TF-IDF vectorizer.
6.2	Fit the vectorizer on the training set.
6.3	Transform the training and testing sets into TF-IDF representations.
7	Train an SVM model:
7.1	Initialize an SVM model with specified parameters
7.2	Fit the SVM model on the training set.
8	Input multiple comments for sentiment prediction.
9	Apply stemming to the input comments.
10	Vectorize the input comments using TF-IDF:
10.1	Transform the stemmed comments into TF-IDF representations.
11	Make predictions on the input comments:
11.1	Predict the sentiments using the trained SVM model.
11.2	Calculate the confidence level of each prediction using the decision function.
12	Display the predicted sentiments and confidence levels for each input comment.
13	End.

Table 10 showcases the external prediction performance of the SVM algorithm using the Porter stemming technique. This table complements the previously mentioned pseudo code in Table 9, which outlines the implementation steps of SVM for sentiment prediction on external Bangla and English text.

**Table 10:** External Prediction performance of Support Vector Machine using porter stemming.

Bangla	English
Comment: দয়া করে আপনারা কিনেন না Sentiment: Negative Confidence Level: 0.79	Comment: Fake products and fake goods Sentiment: Negative Confidence Level: 0.99
Comment: দাম অনুযায়ী ভাল পেয়েছি Sentiment: Positive Confidence Level: 1.00	Comment: The thing is real. Super fast delivery. Sentiment: Positive Confidence Level: 0.99
Comment: দারাজের মত অনলাইন শপ আমরা চাই না বাংলাদেশে Sentiment: Negative Confidence Level: 1.00	Comment: Things are not durable Sentiment: Negative Confidence Level: 42.18
Comment: দারাজের সেবা পেয়ে আমি সন্তুষ্ট নই Sentiment: Positive Confidence Level: 0.36	Comment: The picture has no resemblance to the product Sentiment: Positive Confidence Level: 0.33
Comment: ধুরু, অমাকে হয়রানি করলেন Sentiment: Negative Confidence Level: 1.00	Comment: The quality of the product is good, I'm satisfied Sentiment: Positive Confidence Level: 0.99

Among the studies listed in Table 11, our study stands out as the best performer in terms of sentiment prediction accuracy for both Bangla and English text. While the previous works achieved accuracies ranging from 44.20% to 79.3%, our study achieved an impressive accuracy of 86.43% for Bangla and 82.56% for English text using the Porter stemming algorithm with SVM. Compared to other approaches, such as SentiWordNet, SVM, LSTM, VAE, and various combinations of models and techniques, our study consistently outperformed them in terms of accuracy. The accuracy scores attained by our study surpassed those of previous research by a significant margin.

**Table 11:** Comparison with others state of art studies.

Paper	Dataset	Method & Techniques	Results
Das et al. [49]	2234 sentences	SentiWordNet	ACC 47.6%
Das et al. [50]	447 sentences	SVM	P 70.0%
Tripto et al. [51]	YouTube comments	LSTM and CNN	65.97% (3C), 54.2% (5C)
Ashik et al. [52]	Bengali News comments	LSTM	ACC 79.3%
Palash et al. [53]	Newspaper comments	VAE	53.2%
Hossain et al. [54]	50,000 news, among them 8500 annotated	Traditional linguistic features, SVM, LR, CNN, BERT	Linguistic features + SVM F1-score: 91%. CNN (average pooling): F1-score 59%, CNN (global max): F1-score 54%. BERT model: F1-score 68%
Islam et al. [55]	Facebook comments (2,500)	naïve B	Naive Bayes with Unigram, F-sconaïve.65; Naive Bayes with Bigram, F-score: 0.77
Mahtab et al. [56]	ABSA Bangla dataset	SVM	Accuracy: 73.490%
Sarkar et al. [57]	SAIL dataset of Bangla tweets (1500)	CNN, DBN	CNN Accuracy: 46.80%; DBN Accuracy: 43%
Mandal et al. [58]	Raw Twitter data	Hybrid model combining SGDC and rule-based method.	Language tagging accuracy: 81%; Sentiment tagging accuracy: 80.97%, F-score: 81.2%.
Sarkar et al. [59]	Bangla tweet dataset (SAIL content 2015)	Multinomial naive Bayes, SVM	Multinomial Naive Bayes: 44.20% accuracy; SVM: 45% accuracy
Our Study	Collected 2577 reviews both Bangla and English	Stemming Algorithm: Porter SVM	English: 82.56% Bangla: 86.43%

## 5 Recommendations and policy implications

Based on the findings and contributions of this research paper on sentiment analysis in multilingual contexts, the following recommendations and policy implications can be suggested.

I. Dataset Expansion: To enhance the robustness and generalizability of sentiment prediction models, future research should focus on expanding the dataset size and including a wider variety of sources. This would ensure a more comprehensive representation of sentiment patterns in both Bangla and English text data.
II. Exploration of Alternative Techniques: While the Porter stemming algorithm and SVM models demonstrated effective performance in this study, researchers should explore alternative stemming algorithms, machine learning models, and deep learning architectures. Comparative studies between different techniques would provide insights into their respective strengths and weaknesses, enabling the selection of the most appropriate approach for sentiment analysis.
III. Domain-specific Analysis: Extending the scope of sentiment analysis beyond reviews to different domains, such as social media, news articles, or customer feedback, would be beneficial. Investigating how sentiment prediction models perform across diverse text genres would provide valuable insights for domain-specific sentiment analysis and enable tailored approaches.
IV. Aspect-based Sentiment Analysis: This study focused on overall sentiment prediction; however, research in the future should focus on fine-grained sentiment analysis or aspect-based sentiment analysis. Analyzing sentiment at a more granular level, specifically identifying sentiments towards specific aspects or features, would provide deeper insights into text data and enable more targeted sentiment analysis techniques.
V. Practical Applications: The findings of this research have implications for practical applications in industries such as e-commerce, customer feedback management, and social media monitoring. Organizations can leverage sentiment analysis models to extract valuable insights from customer reviews and improve their products, services, and customer satisfaction.
VI. Policy Development: Policymakers can utilize sentiment analysis techniques to gauge public sentiment towards specific policies, initiatives, or social issues. By analyzing sentiment in multilingual contexts, policymakers can make informed decisions, address concerns, and improve governance strategies.

## 6 Study limitations and scope for future research

While our study on sentiment prediction for Bangla and English text using the Porter stemming algorithm and SVM achieved impressive accuracy results, to acknowledge specific limitations and pinpoint potential avenues for future research is of paramount importance. The scope of our study was limited to a specific dataset comprising 2577 reviews in both Bangla and English. Although this dataset provided valuable insights and yielded promising results, it may not fully represent the diverse range of sentiment patterns present in all Bangla and English text data. Future research could expand the dataset size and include a wider variety of sources to enhance the robustness and generalizability of the sentiment prediction model. Our study focused primarily on the use of the Porter stemming algorithm and SVM. While these techniques proved to be effective in our study, there are other stemming algorithms, machine learning models, and deep learning architectures that could be explored to further improve sentiment prediction accuracy. Comparative studies between different algorithms and models would provide valuable insights into their respective strengths and weaknesses. Additionally, our study primarily considered sentiment prediction in the context of reviews. Future research could explore sentiment analysis in different domains, such as social media, news articles, or customer feedback, to understand how the model's performance varies across diverse text genres. This research solely focused on sentiment prediction, without delving into aspects such as aspect-based sentiment analysis or fine-grained classification of sentiment. Investigating these aspects would enable a more nuanced understanding of sentiment in text data and could lead to more specialized sentiment analysis techniques.

## 7 Conclusion

This research paper made significant contributions to sentiment analysis in multilingual contexts, specifically focusing on English and Bangla languages. The study successfully developed a comprehensive dataset for sentiment analysis in Bangla by collecting reviews in Bengali and their corresponding English translations. This dataset contributes to the availability of resources for sentiment analysis in Bangla. A comparative analysis was conducted, evaluating seven traditional machine learning models and deep learning-based models, implemented four unique neural network architectures, specifically tailored for modelling sequential data in Bengali text analysis. These architectures include LSTM, Bi-LSTM, Conv1D, and a hybrid Conv1D-LSTM. The findings showed that Support Vector Machine (SVM) models exhibited superior performance compared to other models, achieving high accuracies for sentiment analysis in both English and Bangla text using the porter stemming algorithm. The Bi-LSTM Based Model demonstrated the best performance among the deep learning models. The findings offer valuable insights for researchers and practitioners in sentiment analysis, particularly in the context of multilingual sentiment analysis.

## Author contribution statement

Rajesh Kumar Das: Performed the experiments; Analyzed and interpreted the data.

Mirajul Islam: Conceived and designed the experiments; Wrote the paper.

Md. Mahmudul Hasan: Mocksidul Hassan: Sultana Razia: Contributed reagents, materials, analysis tools or data; Wrote the paper.

Sharun Akter Khushbu: Performed the experiments; Contributed reagents, materials, analysis tools or data.

## Data availability statement

Data will be made available on request.

## Declaration of competing interest

The authors declare that they have no known competing financial interests or personal relationships that could have appeared to influence the work reported in this paper.
