# Corrigendum to “Sentiment analysis in multilingual context: Comparative analysis of machine learning and hybrid deep learning models” [Heliyon Volume 9, Issue 9, September 2023, Article e20281]

**DOI:** 10.1016/j.heliyon.2025.e43243

**Published:** 2025-03-26

**Authors:** Rajesh Kumar Das, Mirajul Islam, Md Mahmudul Hasan, Sultana Razia, Mocksidul Hassan, Sharun Akter Khushbu

**Affiliations:** a Department of Computer Science and Engineering, Daffodil International University, Dhaka, 1341, Bangladesh; b Faculty of Graduate Studies, Daffodil International University, Dhaka, 1341, Bangladesh

In this article, references [1] and [2] were included in error:

*[1] S. Kaur Chatrath, G.S. Batra, Y. Chaba, Handling consumer vulnerability in e-commerce product images using machine learning, Heliyon 8 (9) (2022),* https://doi.org/10.1016/j.heliyon.2022.e10743 *.*

*[2] V. Balakrishnan et al., A deep learning approach in predicting products’ sentiment ratings: a comparative analysis, J. Supercomput. 78 (5) (2021) 7206,* https://doi.org/10.1007/s11227-021-04169-6.–7226 *.*

The correct version should be as below:

*[1] Al Tamer M. The advantages and limitations of e-commerce to both customers & businesses. BAU Journal-Creative Sustainable Development. 2021; 2(2):6.*

*[2] Jílková P, Králová P. Digital consumer behaviour and ecommerce trends during the COVID-19 crisis. International Advances in Economic Research. 2021 Feb; 27(1):83–85.*

The authors apologize for the errors.

## Declaration of competing interest

The authors declare that they have no known competing financial interests or personal relationships that could have appeared to influence the work reported in this paper.

